# Pioneering evaluation of GaN transistors in geostationary satellites

**DOI:** 10.1038/s41598-022-17179-y

**Published:** 2022-07-28

**Authors:** Hugo Mostardinha, Diogo Matos, Nuno Borges Carvalho, Jorge Sampaio, Marco Pinto, Patricia Gonçalves, Tiago Sousa, Paul Kurpas, Joachim Wuerfl, Andrew Barnes, François Garat, Christian Poivey

**Affiliations:** 1 Instituto de Telecomunicações - Universidade de Aveiro, Campus Universitário de Santiago, 3810-193 Aveiro, Portugal; 2 Laboratório de Instrumentação E Física Experimental de Partículas (LIP), Av. Prof. Gama Pinto 2, 1649-003 Lisbon, Portugal; 3 EFACEC Sistemas de Electrónica, S. A.– Aerospace Activity Manager, R. Eng. Frederico Ulrich, Apartado 3078, 4471-907 Moreira Maia, Portugal; 4 Ferdinand-Braun-Institut, Leibniz-Institut Für Höchstfrequenztechnik GgmbH, Gustav-Kirchhoff-Straße 4, 12489 Berlin, Germany; 5 ESA/ESTEC European Space Research and Technology Center, Keplerlaan 1, P.O. Box 299, 2200 AG Noordwijk ZH, The Netherlands

**Keywords:** Aerospace engineering, Electrical and electronic engineering

## Abstract

In this paper, we present the results of a 6-year experiment in space that studied the effects of radiation in Gallium Nitride (GaN) electronics in geostationary orbit. Four GaN transistors in a Colpitts oscillator configuration were flown in the Component Technology Test-Bed aboard the Alphasat telecommunication satellite. A heuristic analysis was performed by observing the variation in the power output of the oscillators with the total ionizing dose gathered during the mission. The total ionizing dose was measured with a Radiation Sensing Field Effect Transistors (RadFET) placed close to the GaN devices. The experiment showed that GaN is a robust technology that can be used in the space radiation environment of a geostationary orbit. The work presented here starts with a brief introduction of the subject, the motivation, and the main goal. This is followed by the description of the experimental setup, including the details of the oscillator design and simulations, as well as the implementation of the test-bed and the Components Technology Test-Bed. Finally, the results obtained during the 6 years of experience in space are discussed.

## Introduction

In 2012, a consortium constituted by EFACEC, Instituto de Telecomunicações, EVOLEO Technologies, Laboratório de Instrumentação e Física Experimental de Partículas (LIP), and the Ferdinand Braun Institute (FBH) started a project funded by the European Space Agency to develop several experiments onboard the telecommunications satellite Alphasat. The experiments were part of the Technology Demonstration Payload (TDP-8). It included a promising new type of RF transistor for space and military applications based on gallium nitride (GaN). The objective was to verify and explore the possibility of using GaN technology produced in Europe in geostationary satellites. If GaN successfully operates in space conditions, European satellite manufacturers may benefit from having innovative and highly efficient RF power transistors and MMICs working at higher frequencies. In the long term, they could even replace the current TWTA (traveling wave tube amplifiers) and other technologies onboard satellites.

The experiment flew continuously in orbit from 2013 to 2019, and it is the first experiment of GaN on board geostationary satellites in Europe. Provided evidence about the capability of this technology to operate in space and become a viable solution for substituting TWTA in future satellite and space missions (Despite their higher intrinsic consumption, they consume energy with the need for heating resistors.). The space operational robustness was demonstrated by operating the GaN devices in a real operational space radiation environment. Radiation in space is a hazard for all systems that can degrade performance or even permanently disrupt the operation. It is composed of three parts: galactic cosmic rays (GCRs), solar energy particles (SEPs), and trapped particles. The geostationary orbit is highly exposed to all three components. While GCRs comprise a constant low flux of highly energetic protons and heavy ions that can cause single event effects (SEEs), SEPs consists of a very large flux of energetically charged particles emitted from the Sun in stochastic events that can deliver a high total ionizing dose (TID) in a short period of time. The Van Allan belt trapping these particles extends to the geostationary orbit, namely, in the form of an outer electron belt with energies up to ~ 10 MeV, which can penetrate spacecraft shielding and lead to high TID levels^1^.

Although GaN radiation damage studies are still in an early phase, it is known that the main mechanism of radiation degradation is caused by displacement damage from protons and electrons and single event burnout (SEB) when exposed to heavy-ion radiation^2,3,4^. The inherent hardness of GaN Schottky gated devices to TID comes from the fact that metal–oxide–semiconductor (MOS) contacts are not present. The number of traps generated in the vicinity of the gate electrode is therefore decreased. Such traps lead to TID effects on device performance (leakage increase and shift of threshold voltage)^2^. Displacement damage occurs when an incident particle collides with the nucleus of a lattice atom, transferring enough energy to displace it. Displaced atoms may form stable defects or traps, resulting in decreased mobility, threshold voltage shift, decrease in transconductance, and decreased drain saturation current^3^. SEB occurs when an incident particle passes through a high field region in the device and thus induces a localized high-current state, which can lead to catastrophic failure of the device. Electrically conductive filaments may occur, for instance, when heavy ions are impinging through sensitive device regions such as field plates or MIM capacitors^5^. While radiation ground tests are the baseline of radiation hardness assurance of electronic devices, the high stakes of space missions make flight demonstrations a critical part of technology development, especially because no facilities can fully reproduce the space radiation environment and other physical conditions. The experiment presented here aimed to demonstrate the space-borne reliability of GaN devices in the conditions found in geostationary orbit.

The experiment was based on 4 oscillators operated at 2.5 GHz, which were continuously monitored during the mission. Data collected for analysis included drain-source voltage (*Vds* ), gate-source voltage (*Vgs*), drain-source current (Ids), power of the radio frequency (RF) signal generated by the oscillators, energy consumption, temperature, and TID to which the components were subject^6^. TID was measured using a Radiation Sensing Field Effect Transistors (RadFET) at board level (on the oscillator Printed Circuit Board (PCB)). A full description of the TID assessment can be found in^7^. The measured TID for the whole duration of the mission was 3.3 krad.

The complete experiment was designed and realized by the consortium, including the four oscillators that allowed studying the part-to-part variability due to radiation effects. To perform the interconnection and control of all circuits, a motherboard was also designed. This board powers the circuits to control the oscillators measure the characteristic parameters of the oscillators and communicate with the satellite control unit. It allowed independent powering or shutting down of each of the oscillators; it also fed the power detector, measured the oscillator power taking into account the thermal effect of the measurement power detector diodes themselves, and measured the onboard temperature and the radiation level to which it will be exposed. The main components of this board are presented in more detail in the next sections.

### GaN transistors samples

GaN high-electron-mobility transistor (HEMT) devices constitute a very promising technology for high-power applications. Their unique physical properties, such as high bandgap (3.4 eV) and high breakdown field (4 MV/cm), enable the construction of optimized devices for microwave and millimeter-wave applications. As the breakdown capability of the material is high, devices with small source-to-drain distances are feasible. Along with the capability of having a high channel current density, this significantly reduces ohmic losses in the device. Therefore, GaN enables highly efficient high-power RF amplifiers. They outperform their Si, GaAs, and SiC competitors in terms of output power, efficiency, and linearity. Therefore, they are of extreme interest for space applications and enable novel highly innovative systems, such as beam steering concepts, replacement of traveling wave tubes, and many other applications^8^.

**Figure 1:**
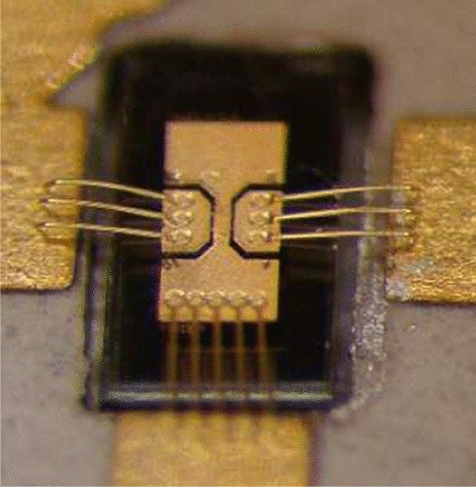
Photograph of the GaN transistor mounted in the oscillator PCB. Chip dimensions are 660 × 210 µm^2^.

The active GaN devices selected for the Alphasat payload experiment were designed, epigrown, and processed at the Ferdinand Braun Institute (FBH) in Berlin in the framework of the European Space Agency (ESA)-funded GaN benchmarking project (Contract No. 20328/06/NL/IA). They consisted of a 2-finger (2 × 50  μm) GaN High Electron Mobility Transistor (HEMT) topology with a total gate width of 100 μm and a gate length of 0.5 μm. The devices were realized on semi-insulating SiC substrates with the corresponding GaN and AlGaN epitaxial layers, optimized for L- to X-band applications. The transistors had a Pt-based Schottky T-Gate metallization structure without field plates and were fully passivated using a SiNx coating. Figure 1 shows a discrete GaN transistor mounted into the oscillator PCB environment. The device periphery has been specially designed to account for rugged and reliable mounting as required for space applications (chip soldering and Au wire wedge bonding, see Fig. 1). Wafer S-parameter probing demonstrated a transition frequency ft=$36\mathrm{ GHz},$ and a maximum frequency of power amplification fmax = 78 GHz at a 28 V drain bias. At a 2 GHz operation frequency, the transistors reached a power density of 6 W/mm.

### GaN control board

**Figure 2:**
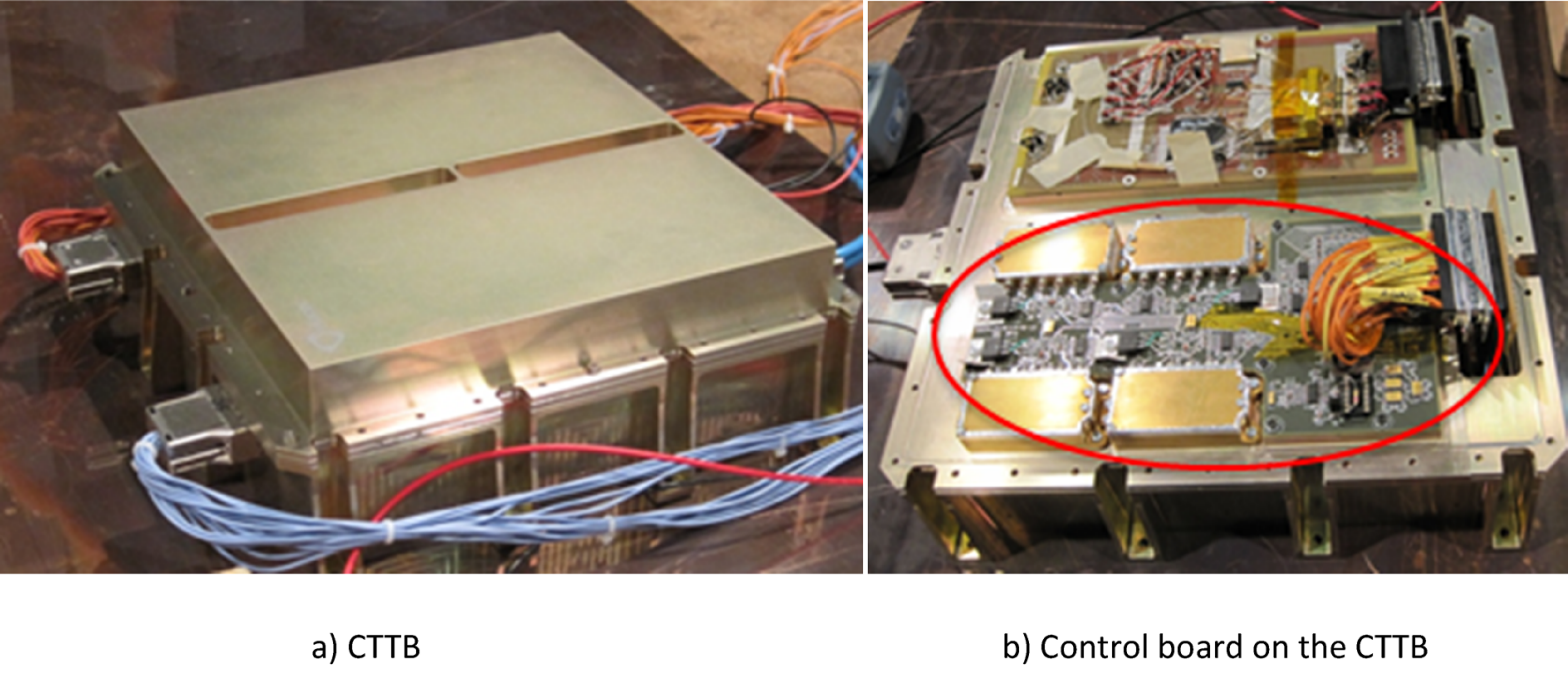
(**a**) CTTB, (**b**) Control board on the CTTB.

**Figure 3:**
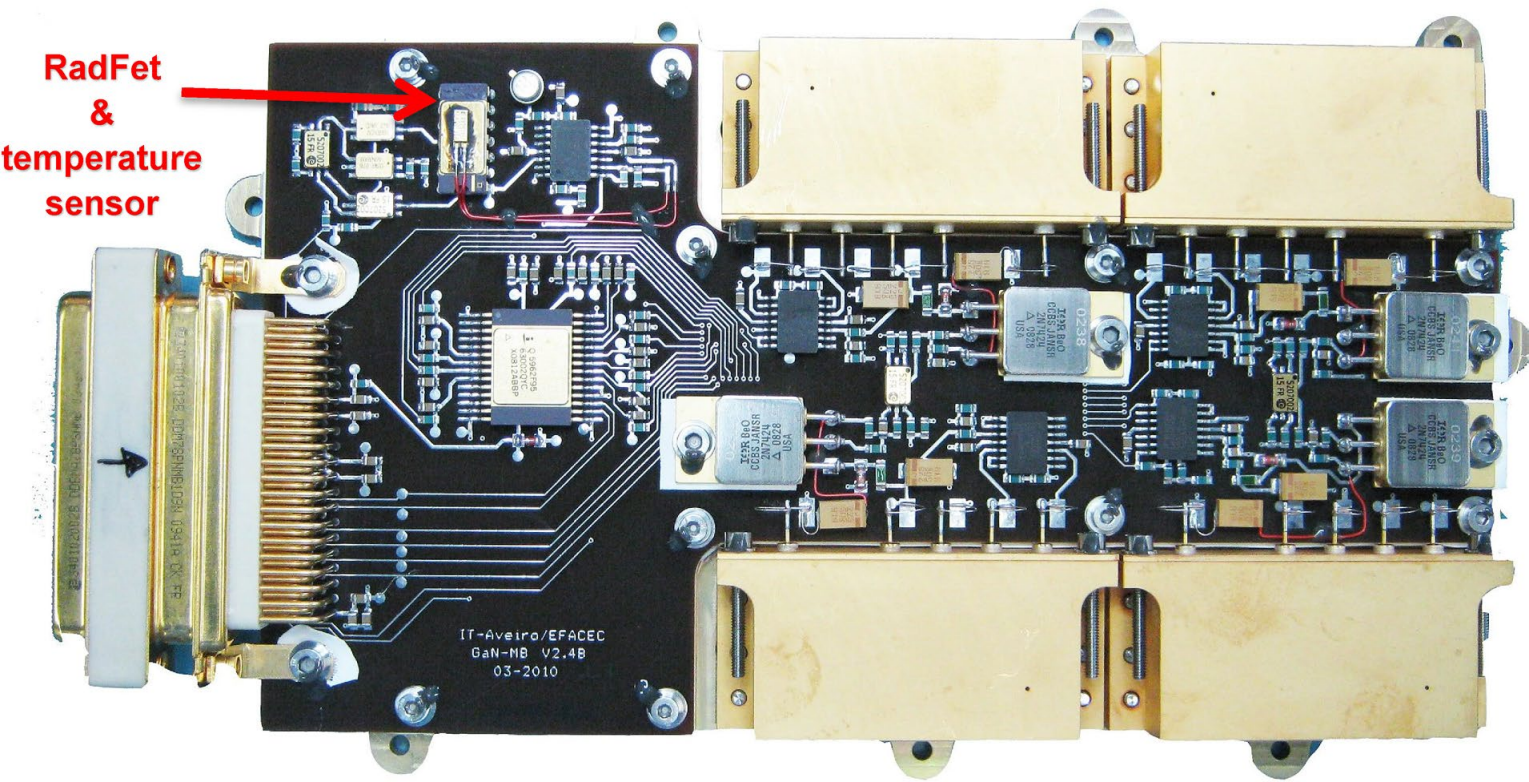
Test breadboard.

A control board (Figs. 2 and 3) was developed with the function of housing the oscillators and sensors to interconnect those sensors with a communication protocol to deliver the data to the internal satellite communication bus and finally transmit them to an earthbound ground station.

The board encompasses electrical current sensors, RF power level sensors, and sensors for measuring, temperature and radiation level; furthermore, the oscillators can be shut down individually. All signals are properly isolated with buffers, and the data of each oscillator are combined into a single pack of information before communicating with the Components Technology Test Bed (CTTB) part that manages the experimental data.

The experiment consisted of three well-defined stages: the first stage consisted of making a functional prototype; the second stage was the realization of the board, including the final oscillators; and finally, the assembly of all the equipment.

#### Circuit selection decision

The first decision was to select which kind of electrical circuit should be used for validating GaN FET devices in space. Several types of circuits were taken into consideration, such as amplifiers, mixers, or oscillators. For example, amplifiers could have been an optimum choice for GaN technology onboard satellites because such devices could potentially replace traveling-wave tube amplifiers (TWTAs) in the future. However, the high values of consumed power, the need for external signal sources and driver circuits for testing, and last but not least, the mass increase for the experiment rendered this option unviable for this experiment. RF oscillators, however, do not need extra signal excitation sources, which allows the inclusion of all circuitry and its measurement systems inside the same box, as well as the reduction of power consumption and mass. For this reason, the circuit selected was the oscillator, since it allows the integration of all the sensors on the same board, optimizing power, mass, and space on the board.

#### Oscillator design and implementation

**Figure 4:**
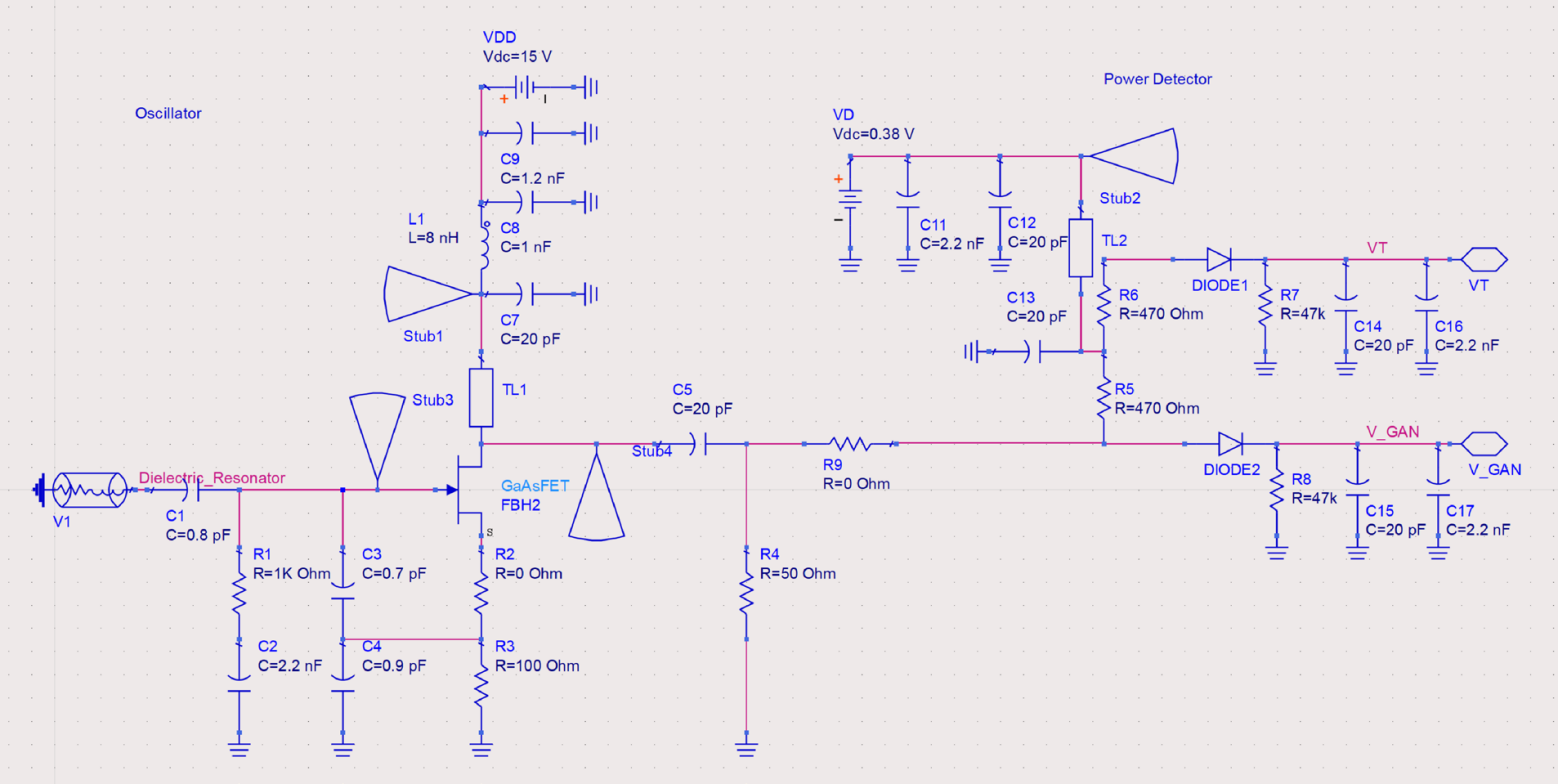
Oscillator schematic.

**Figure 5:**
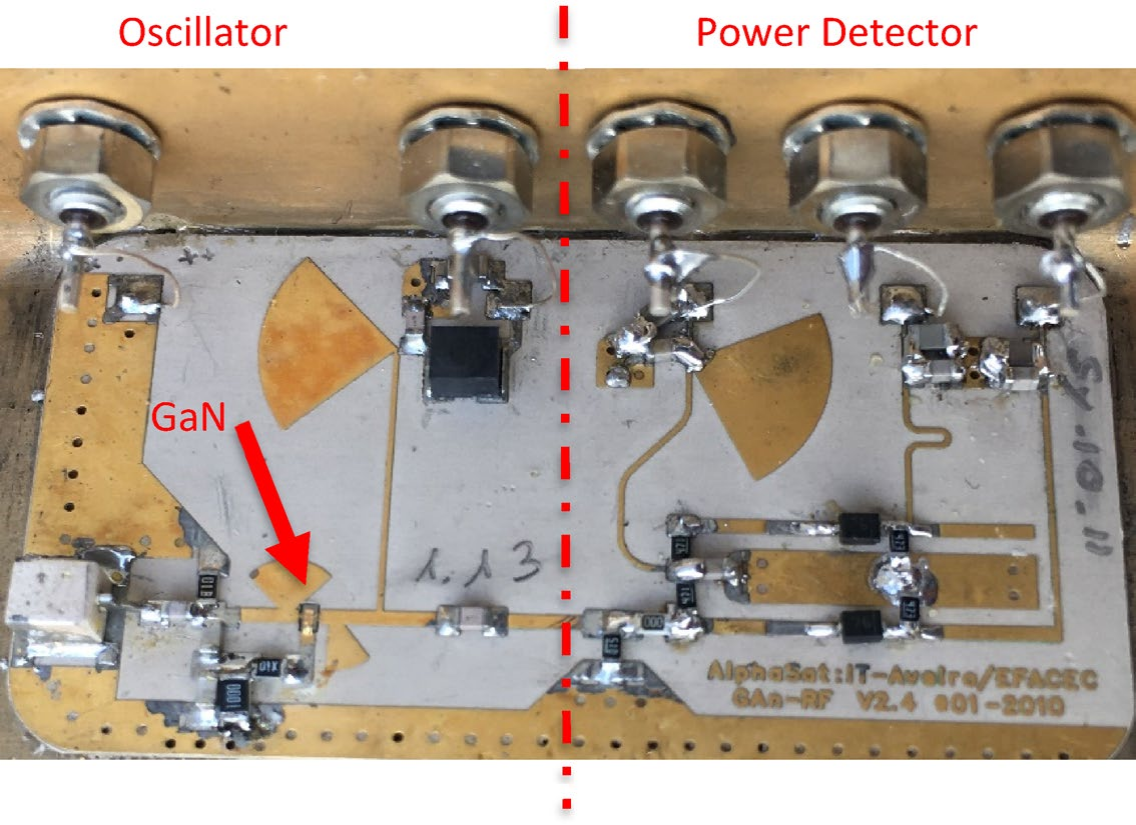
Prototype.

The adopted RF oscillator topology follows a Colpitts configuration and assumes that the feedback loop is made by a capacitive/resistive network. The inductor for the Colpitts arrangement, and thus for the resonance loop, is made by a coaxial resonator. The oscillator schematic can be seen in Fig. 4 and the prototype in Fig. 5.

Since it is a circuit designed to be used in a space environment, moving parts are to be avoided, which limits the use of adjustable components such as variable capacitors. Thus, a coaxial resonator was used, with a precise frequency of operation that subsequently determines the oscillation frequency^8^.

The frequency selected to operate the oscillator was 2.5 GHz, and the nominal *Vds* for the devices could be as high as 28 V, but due to onboard DC power supply availability, the *Vds* voltage had to be limited to 15 V, and the gate voltage was maintained at *Vgs* = 0 V. Operating the GaN devices in an oscillator environment allowed the transistor to be excited hardly and even harder than in a traditional power amplifier^4^.

In addition to the RF oscillator itself, the system test board also included a temperature compensated power detector to monitor the power of the GaN circuit while in space. Thus, a power probe was also developed and included in front of the oscillator. Therefore, the whole configuration was a complete RF laboratory in space. The output of the measurement circuit consists of two DC voltages, one corresponding to the oscillator power and the other to a calibration voltage for temperature calibration.

The oscillator was built using Duroid RT6010 substrate and attached to the external housing case with electrically and thermally conductive adhesive glue (ATI-ESP8350). This had been dispensed in the absence of water to prevent crystallization, which could create ruptures and volume changes. To avoid cracks due to a mismatch of the coefficient of thermal expansion (CTE), the case material had a CTE near one of the substrates as well as good electrical and thermal properties. Furthermore, it needs to be a lightweight and robust material. For all the above-mentioned reasons, the special alloy CE-17 coated with 10 μm Ni + and capped with 1 μm Au was chosen.

**Figure 6:**
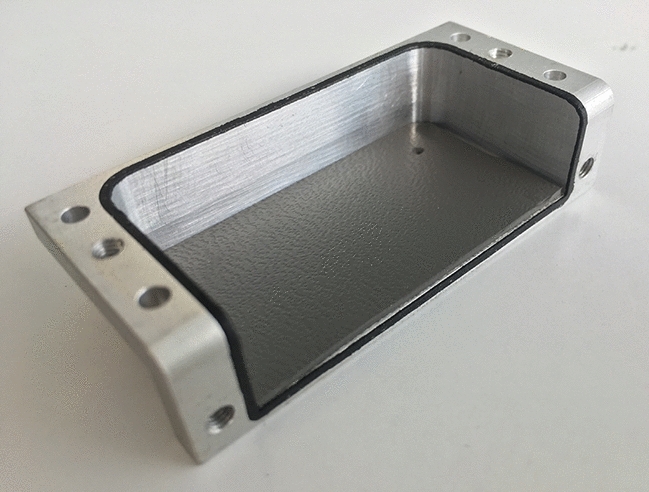
Oscillator enclosure with absorbers.

The arrangement of the oscillator circuit must also guarantee RF isolation up to approximately 40 dB below the generated signal so that there is no electromagnetic interference with the satellites’ own radio systems. Therefore, the oscillator had to operate in a completely encapsulated Faraday cage. To improve the chip thermal dissipation and ground connection, it was glued directly to the box using epoxy glue AIT (EG8050). The upper case is shown in Fig. 6.

In addition, we also used an RF absorber material glued inside the cover lid by using silicone base glue (ECCOSORB BSR-2-SS6 M), as shown in Fig. 6. Thermal and vacuum test cycles were conducted to study the temperature dependence, and the RF signal power isolation was measured in an anechoic chamber.

## Results and discussion

This section summarizes the results obtained from operating the developed boards in a geostationary orbit for 6 years from July 2013 to April 2019. The experiment was intended to monitor the effect of the total ionizing dose (TID) from energetically charged particles and from space temperature cycles on the power output of the GaN oscillators. The figures presented below show the data collected for the whole mission duration.

**Figure 7:**
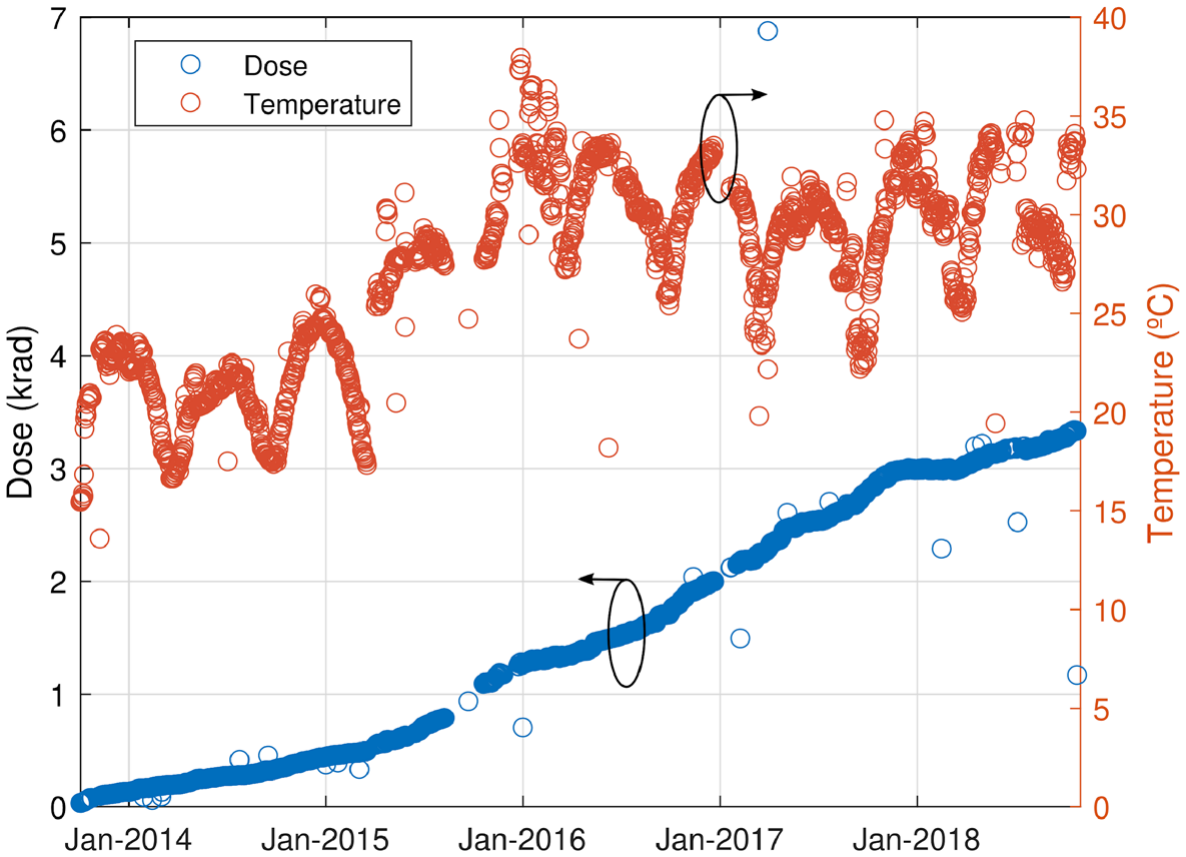
Variation in the temperature and dose over time.

Figure 7 shows the variation of the temperature and dose depending on the time elapsed. Note that initially, the satellite was not 100% functional, where only some parts were working. Thus, from April to October 2015, the satellite started to operate at 100%, which increased the global temperature. This dependency can be seen in Fig. 7. From the same figure, it is possible to observe that the radiation dose increases over time, as expected.

**Figure 8:**
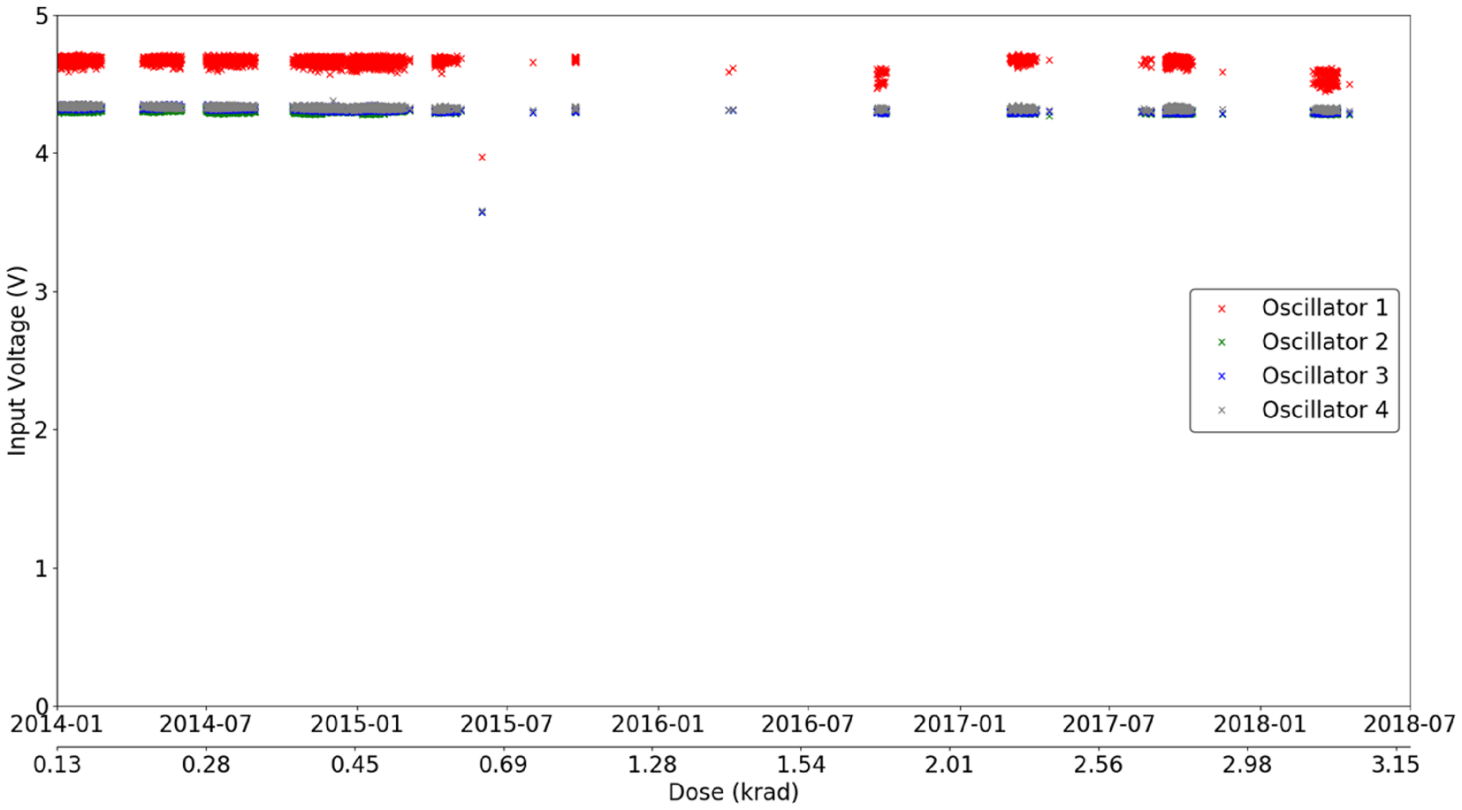
CCTB Input voltage of all oscillators as a function of time and dose for temperatures between 22 and 25 °C.

Figure 8 presents the relation between each oscillator regarding its output power and the radiation dose and temperature. In general, it appears that the output power slightly increases when the system temperature cools down, which is expected behavior^8^. Another relevant aspect is that the output power decreases with increasing radiation dose, albeit slightly, which leads to the conclusion that the aging process and radiation cause small changes in the device’s behavior. All four oscillators present the same pattern, oscillator 4 with 12%, and the other three oscillators have a variation of approximately 10% of their output power.

The power output of the oscillators is a function of the input voltage (the result of the oscillator output power level with internal thermal correction), temperature, and, possibly, the total ionizing dose (TID). Figure 7 shows the variation in the CCTB board temperature (temperature sensor is located over the RadFet sensor) and dose over time. Fluctuations in temperature are caused by the Earth’s orbit around the Sun. The large increase in the average temperature in 2015 is related to satellite operations that were only partly on up to this date. Since the power output is highly sensitive to the temperature, only the measurements taken inside a small range of temperatures between 22 and 25 °C, which was reached during the whole mission for short periods of time, were considered for the analysis. The input voltage was fairly stable during the whole mission for these temperature values, as shown in Fig. 8. For this reason, one can assume that the power output did not change with the input voltage for this analysis for each oscillator.

**Figure 9:**
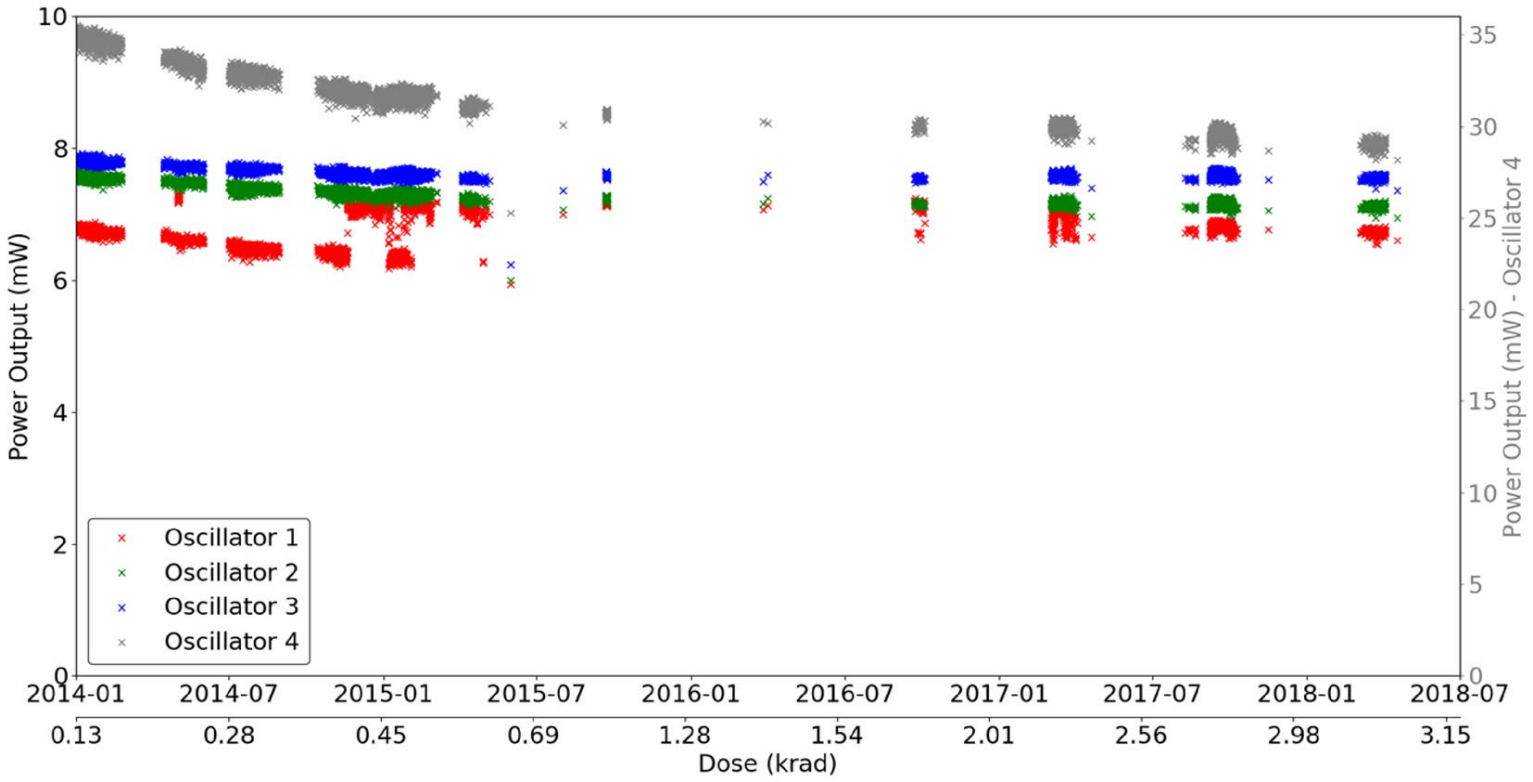
Power output of all oscillators as a function of time and dose for temperatures between 22 and 25 °C. Only Oscillator 4 shows a significant decrease in power output. (In this case, the transistors have not gone through a combined thermal/electrical aging process, so it is expected that the first part of the graph corresponds to a burn-in effect).

Considering the measurements taken in a temperature interval between 22 and 25 °C, the power output for all four oscillators is shown in Fig. 9. Three out of four Oscillators reach a stable operation level on orbit, note that Oscillator 4 shows an approximately 12% output power decrease during the first year of operation, due to a known higher compressed bias point in the manufacturing process. On the other hand, this particular oscillator also had a higher initial power output. It should be noted that all transistors were subjected to a thermal burn-in procedure after manufacturing and selected such that their DC performances were comparable. We assume that the differences in the individual oscillators are due to local variations of dispersive effects on the particular wafers, which lead to slightly different initial power performances, although the DC data are quite similar. At the time when the GaN devices were provided for these space experiments, no biased burn-in procedures were undertaken. This could of course have reduced the observed variabilities. Nevertheless, all oscillators remained active until the end of the mission with small variations in their power output, see Fig. 9. It is important to consider this result in light of the radiation environment to which the devices were subject. Geostationary orbit is populated mostly by electrons, which leads to high TID but low displacement damage dose. The fact that GaN technology is known to be more susceptible to the latter and the fact that the measured TID was not very high (~ 3.3 krad) explains why no noticeable damage to the components was measured.

## Conclusions

In this paper, we presented the results from a 6-year experiment aboard the Alphasat in Geostationary orbit to study the behavior of GaN technology when subject to the space radiation environment. Four GaN transistors in a Colpitts oscillator configuration were mounted on a board of the CTTB instrument together with a temperature sensor circuit and a RADFET for TID assessment.

Both the oscillator power output and the input voltage were monitored for four oscillators. The results showed negligible variation in three of the oscillators. The fourth oscillator’s power output decreased by approximately 12% during the first year of operation. Nevertheless, all oscillators were performing within specification for the whole duration of the experiment. From these results, the experiment provided evidence supporting the reliability of GaN technology in future space missions, at least in geostationary orbit considering that this is an electron-rich orbit with a reasonable total ionizing dose (TID) and low displacement damage dose, which is ideal for a technology that has shown low sensitivity to the former.
